# Electrocardiographic Predictors of Cardiovascular Mortality

**DOI:** 10.1155/2015/727401

**Published:** 2015-07-16

**Authors:** Ioana Mozos, Alexandru Caraba

**Affiliations:** ^1^Department of Functional Sciences, Victor Babes University of Medicine and Pharmacy, 300173 Timisoara, Romania; ^2^1st Department of Internal Medicine, Victor Babes University of Medicine and Pharmacy, 300173 Timisoara, Romania

## Abstract

Cardiovascular diseases are the main causes of mortality. Sudden cardiac death may also appear in athletes, due to underlying congenital or inherited cardiac abnormalities. The electrocardiogram is used in clinical practice and clinical trials, as a valid, reliable, accessible, inexpensive method. The aim of the present paper was to review electrocardiographic (ECG) signs associated with cardiovascular mortality and the mechanisms underlying those associations, providing a brief description of the main studies in this area, and consider their implication for clinical practice in the general population and athletes. The main ECG parameters associated with cardiovascular mortality in the present paper are the P wave (duration, interatrial block, and deep terminal negativity of the P wave in V1), prolonged QT and Tpeak-Tend intervals, QRS duration and fragmentation, bundle branch block, ST segment depression and elevation, T waves (inverted, T wave axes), spatial angles between QRS and T vectors, premature ventricular contractions, and ECG hypertrophy criteria.

## 1. Introduction

Cardiovascular diseases are the main causes of mortality and an important public health problem. Sudden cardiac death is a major health problem, and most of the cases occur outside the hospital, without warning signs [1, 2]. Structural and functional cardiovascular abnormalities act as predisposing factors to cardiac arrest, with a sudden onset of electrical instability with ventricular fibrillation, not preceded by previous symptoms [3].

Despite the benefits of physical activity, vigorous exertion may also increase the risk of sudden cardiac death and other acute cardiovascular events [4]. Sudden cardiac death, often the first clinical manifestation of a cardiovascular disorder, is more prevalent in older athletes (older than 35 years) due to atherosclerotic coronary heart disease [4, 5]. Congenital or inherited cardiac abnormalities, including hypertrophic, dilated, or Phidippides cardiomyopathy and coronary arteries anomalies, premature atherosclerosis, myocarditis, Marfan syndrome, valvulopathies, preexcitation syndromes, or cardiac channelopathies, are the main causes of acute cardiovascular events in younger athletes [3–7], and physical exercise acts as an arrhythmogenic trigger [8]. Cardiovascular screening cannot identify all athletes at risk; however, some signs are visible on the electrocardiogram (ECG), despite silent cardiovascular abnormalities. Several subgroups have a higher cardiovascular risk, especially males, older individuals, Afro-Caribbean descendants, basketball players, joggers or marathon racers, and endurance athletes [5, 9]. In middle-aged and older individuals, vigorous exertion increases the risk of coronary events in those who did not exercise regularly, but habitual physical activity reduces the incidence of sudden cardiac death and myocardial infarction [5].

Physiological cardiovascular adaptation to sustained physical exertion, “athlete's heart,” causes several common ECG changes, not associated with increased cardiovascular risk, such as sinus bradycardia, due to increased parasympathetic tone, first degree atrioventricular block, early repolarization, incomplete right bundle branch block, and increased QRS voltage due to physiologic left ventricular hypertrophy [5, 9]. Several recommendations for the interpretation of ECG in athletes were elaborated, in order to eliminate false-positive ECG results: the recommendations of the European Society of  Cardiology [10], the “Seattle criteria” [11], and, lately, the “Refined Criteria for ECG Interpretation” [12, 13]. Besides the training-related normal findings, borderline changes were also described and training-unrelated abnormal findings [13].

The electrocardiogram, the written record of a heartbeat, is used in clinical practice for diagnosis and prognosis, but also in clinical trials, as a valid, reliable, repeatable, quantitative method, inexpensive and unbiased by clinical information [14].

**Figure 1:**
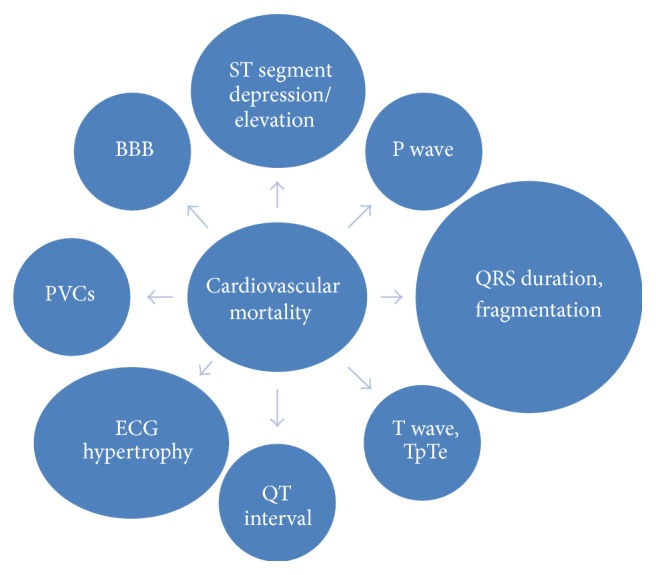
Electrocardiographic predictors of cardiovascular mortality. TpTe = Tpeak-Tend interval, PVCs = premature ventricular contractions, and BBB = bundle branch block.

The aim of the present paper was to review ECG signs associated with cardiovascular mortality and the mechanisms underlying those associations, providing a brief description of the main studies in this area, and consider their implication for clinical practice in the general population and athletes. Several ECG variables are associated with cardiovascular mortality (Figure 1).

## 2. P Wave

The P wave is the expression of atrial depolarization and atrial conduction. P wave indices derived from the standard ECG are P wave duration, morphology, and amplitude and provide information about the atrial electrical activity.* Prolonged P wave duration* signifies a conduction delay between the left and right atria, called* interatrial block*, and is related with atrial fibrillation and, lately, with increased all-cause cardiovascular disease and stroke mortality in the general population [15–17]. Coronary artery disease and traditional cardiovascular risk factors, including hypertension, hypercholesterolemia, smoking, obesity, sedentary lifestyle, and higher age, have been associated with interatrial block, probably due to endothelial dysfunction and ischemia-related interatrial conduction delay [18]. Interatrial block was also reported in patients with left atrial enlargement and electromechanical dysfunction, lower left atrial stroke volume, and kinetic energy, which could explain excess stroke mortality enabling atrial thrombosis [17, 19, 20].

During a follow-up of 18 years in a study including 739 middle-aged type 2 diabetic patients without previous major cardiovascular events, prolonged P wave duration was associated with increased stroke mortality among patients with nonmajor cardiovascular disease, independent of conventional cardiovascular risk factors, proteinuria, duration and treatment of diabetes, glycemic control, heart rate, and left ventricular hypertrophy [17].

*Deep terminal negativity of P wave in V1* (DTNPV1), a marker of atrial abnormality, defined as biphasic P wave, with the negative P prime larger than 1 mm, easily detected by simple visual inspection of the resting 12-lead ECG, was associated with an increased risk of death due to all-cause cardiovascular disease and ischemic heart disease in a large study, including 8,146 participants, during a follow-up of 13.8 years [21, 22]. DTNPV1 was also related to an increased risk of sudden cardiac death, but also to an increased risk of nonfatal events (atrial fibrillation, coronary heart disease, heart failure, and stroke) in the 15,375 participants of the ARIC (Atherosclerosis Risk in Communities) study, after 14 years of follow-up [22].

To conclude, the most important atrial ECG predictors of cardiovascular death are prolonged P wave duration, interatrial block, and deep negativity of the P wave in V1.

*Left and right atrial enlargements* are considered minor abnormal findings according to the “Refined Criteria for ECG Interpretation” for elite athletes [13] and are infrequent in both athletes and patients with cardiomyopathy [9]. Left atrial enlargement coexists with other ECG changes in hypertrophic cardiomyopathy, such as T wave inversion, Q waves, and ST segment depression [13].

## 3. QT Interval

The QT interval, from the onset of the QRS complex to the end of the T wave, is a reflection of the summed ventricular action potential durations in the heart [23]. The relation between prolonged QT intervals and adverse cardiovascular events has been the aim of several studies. The QT interval has limitations, such as difficulties in delineating the end of the T wave, diurnal variation, variability due to method and technique, and intraobserver and interobserver variability. Heart rate correction is another major problem of the QT interval, considering that the Bazett formula, most frequently used in clinical practice and research, overcorrects at high heart rates and undercorrects at lower heart rates and is appropriate for physiological heart rates only. The relation between QT interval and HR seems to be individualized [24]. The QT interval has several sources of variability, including advanced age, gender, drugs, body mass index, autonomic changes, diabetes mellitus, dyslipidemia, smoking, heart failure, myocardial ischemia, hypertension, stroke, impaired renal function, liver cirrhosis, and electrolyte imbalances [25]. Accurate determination of the end of the T wave may be difficult especially in flat, bizarre, or other abnormal patterns of T waves. Heart rate corrected QT interval duration (QTc) predicted sudden cardiac arrest in the participants of the Prediction of Arrhythmic Events with Positron Emission Tomography (PAREPET) study, with depressed left ventricular ejection fraction and ischemic cardiomyopathy [26].

Torsade de pointes do not appear in some patients with prolonged QT intervals, if the transmural dispersion of repolarization is not increased or if the repolarization reserve is normal [27]. More sensitive predictors of cardiovascular events are needed.

A heart rate corrected QT interval of 470 ms or longer is considered a training-unrelated abnormal finding in elite athletes [13]. Not just long but also short QT intervals are considered training-unrelated ECG abnormalities in athletes [9].

## 4. QRS Duration and Fragmented QRS

QRS duration predicts mortality in patients with left ventricular dysfunction and hypertension [28] and cardiovascular mortality in the general population, according to a study enrolling 46,933 patients, with a mean follow-up of 6 years [29]. Any 10 ms prolongation of the QRS duration caused an 18% increase of the cardiovascular risk [29].

Development of fragmented QRS is related to intraventricular conduction delays due to inhomogeneous activation of the ventricles caused by myocardial scars, myocardial ischemia and fibrosis, impaired signal transduction and ventricular depolarization, and peri-infarction conduction blocks [30–34]. Fragmented QRS was shown to predict major cardiac events, death, larger myocardial infarct size, and low left ventricular ejection fraction in patients with acute coronary syndromes in a study enrolling 355 patients, hospitalized in a coronary intensive care unit [33]. Not only the presence of fragmented QRS but also the number of leads with fragmented QRS counts [33, 35]. The presence of three or more leads with fragmented QRS was found as an independent predictor of cardiac death or hospitalization for heart failure in patients with prior myocardial infarction [35]. Fragmented QRS in the presence of a wide QRS, exceeding 120 ms, is also a sign of myocardial scar and an independent predictor of mortality in patients with coronary heart disease, according to a study including 879 patients, during a follow-up of 29 months [32]. The possible mechanisms explaining adverse outcomes in patients with fragmented wide QRS could be scar related development of heart failure, coronary, or arrhythmic events [32]. Fragmented QRS predicted mortality not only in patients with coronary heart disease, but also in those with implantable cardioverter defibrillator, nonischemic cardiomyopathy, arrhythmogenic right ventricular cardiomyopathy, and Brugada syndrome and in patients undergoing coronary artery bypass graft surgery [34, 36].

Various types of fragmented QRS were described in the literature, including notches on the R or S wave, RSR′ pattern, additional R waves, fragmented right and left bundle branch block, and fragmented QRS associated with ST segment elevation [31, 33, 34].

The rSr′ pattern in leads V1-V2 can be found in benign or sever life-threatening heart diseases, including the Brugada syndrome or arrhythmogenic right ventricular dysplasia. It was frequently associated with syncope or cardiac arrest, and it also appeared in healthy, asymptomatic young persons and athletes or when leads V1 and V2 were placed higher [37]. A R′ pattern in leads V1 and V2 may also be related to right ventricular enlargement, some cases of ventricular preexcitation, and hyperkalemia [37].

Strauss et al. [38, 39] used a QRS score including Q, R, and S wave amplitudes, durations, and notches in 10 of the 12 standard ECG leads, identifying and quantifying scars in ischemic and nonischemic cardiomyopathy patients and concluding that patients with no scar by QRS scoring have significantly fewer ventricular arrhythmia.

Notched QRS in V1 is included in the common, training-related ECG abnormalities in athletes, but Brugada-like early repolarization is a major, training-unrelated ECG abnormality in athletes [9].

In other words, fragmented QRS complexes predict cardiovascular mortality in patients with left ventricular dysfunction, acute coronary syndromes, Brugada syndrome, and arrhythmogenic right ventricular dysplasia, but not in athletes.

## 5. Bundle Branch Blocks (BBB)

Aggressive therapy of myocardial infarction using thrombolytic agents and early coronary revascularization reduced the incidence of Q wave myocardial infarction and increased non-Q myocardial infarction, making the diagnosis of an old myocardial infarction in the presence of bundle branch block more difficult [32, 40]. Late notching of the S wave in at least two leads (V1–V4), Q waves in at least two leads (I, aVL, V5, and V6), R wave regression from V1 to V4, and primary STT changes in at least two adjacent leads were demonstrated to be signs of myocardial infarction in the presence of a complete left bundle branch block [41]. A* new left bundle branch block* could predict an acute myocardial infarction with a sensitivity of 42% and a specificity of 65% in 182 patients with left bundle branch block (13% with an acute myocardial infarction) [42]. Notching of the S wave in addition to a notched R wave in left bundle branch block was named “*fragmented left bundle branch block,*” a significant sign of myocardial infarction scar and mortality [32].* Right bundle branch block* may represent a myocardial scar located in the right ventricle or inferior wall, with a better prognosis than a left bundle branch block [32]. A left bundle branch block pattern with a low amplitude of the QRS complex could be due to uncoupling in the left ventricular working myocardium, caused by reduced expression or misalignment of connexins during left ventricular hypertrophy, and cardiac resynchronization therapy could be detrimental in those patients [43].

Complete left BBB or right BBB are not related to training in elite athletes and are considered abnormal findings [13]. On the other hand, an incomplete right BBB and increase of QRS voltages may be due to an increased ventricular mass (left or right), due to increase of both cavity dimensions and wall thickness, and are included in the physiological electrocardiographic changes in the athlete's heart [9]. A complete right or left bundle branch block is a training-unrelated ECG abnormality in athletes [9].

To conclude, a left bundle branch block could predict an acute myocardial infarction and right bundle branch block a myocardial scar in the right ventricle. Both complete left and right bundle branch blocks are abnormal ECG findings in athletes.

## 6. ST Segment Depression or Elevation

*ST segment elevation* has been described in many conditions, including acute myocardial infarction, Brugada syndrome, early repolarization, and acute pericarditis [1]. Elevated ST height at J point (STJ) in leads V3, II, and aVF and ST height at 60 ms after the J point (ST60) were “protective” from sudden cardiac death in the combined cohorts of the ARIC study and CHS (cardiovascular health study) [1]. STJ and ST60 were tested in individual leads, considering that different ion channels may be active only in some leads [1]. ST segment elevation is highly prevalent in all black individuals irrespective of athletic training and suggests an ethnicity-related effect [44].

Isolated minor nonspecific ST segment and T wave abnormalities, such as* upsloping ST segment depression* and flat or inverted T waves, are common in asymptomatic, older patients and were significantly associated with an increased risk of coronary death and primary arrhythmic death in a more than a decade follow-up study [45]. Nonspecific ST segment and T wave abnormalities have been related to cardiovascular disease and coronary heart disease mortality in middle-aged persons [46–48]. It was suggested that nonspecific ST segment and T wave abnormalities might signify subclinical coronary disease or left ventricular hypertrophy [47], but in the CHS, adjustment for subclinical atherosclerosis and left ventricular mass did not influence the association between the STT changes and cardiovascular endpoints, suggesting that arrhythmias may be involved [45]. Isolated minor nonspecific STT abnormalities have been also associated with physiologic phenomena, such as ingestion of food, postural changes, emotional stress, hyperventilation, or central nervous system injuries, abnormalities in left ventricular wall motion, electrolyte disturbances, use of drugs or athletic abilities, and heart failure, but none of these could explain the association with fatal cardiovascular events [45]. It seems that persistent, nonspecific STT changes, and not those due to transient physiological phenomena, are significantly associated with cardiovascular mortality [45, 49].

Regular, intensive exercise is associated with repolarization changes affecting the ST segment and T wave morphology [44]. ST segment depression and Brugada-like pattern, especially if associated with pathological Q waves and T wave inversions beyond V1 in Caucasian athletes, are training-unrelated abnormal findings in athletes [13]. On the other hand, early repolarization, together with sinus bradycardia and first and second degree atrioventricular block occur due to an increased vagal tone and/or withdrawal of the sympathetic activity as physiological electrocardiographic changes in the athlete's heart (group 1 or common training-related ECG abnormalities in athletes) [9]. The STJ/ST80 ratio (ST segment elevation at J point/ST segment at 80 ms after the J point) may help in differentiating early repolarization from Brugada syndrome in athletes [50]. A downsloping ST segment configuration (STJ/ST80 > 1) was found in patients with Brugada syndrome and an upsloping ST-configuration (STJ/ST80 < 1) showed high diagnosis accuracy for early repolarization [50].

In other words, ST segment depression predicts coronary and arrhythmic death and is an abnormal finding in athletes.

## 7. T Wave

There is limited information about the utility of repolarization subintervals. The* Tpeak-Tend interval (TpTe)* is an index of transmural dispersion of repolarization, a marker of ventricular arrhythmia vulnerability [51, 52]. The most important limitation of the TpTe is lack of a clear consensus about normal values.

*Inverted T Wave and T Wave Axes*. Abnormally inverted T wave and prolonged QTc, increased heart rate, and hypertension were found to be stronger predictors of sudden cardiac death risk than coronary heart disease in a study enrolling 18,497 participants, initially free of coronary heart disease, after a follow-up of at least 13 years [1]. T wave inversion, QRS duration, and QRS/T angle were significantly associated with the risk of sudden cardiac death and death from all causes, beyond conventional cardiovascular risk predictors in the general population, according to a study including 1,951 men, followed up for 20 years [53].

According to a recent consensus article, negative T waves are not caused by acute ischemia but appear in chronic or vanishing ischemia, and the negative ischemic T waves are symmetrical, of variable deepness, presenting mirror patterns, and may be accompanied by positive or negative U waves [54]. Myocardial edema rather than systolic dysfunction underlies the Wellens' ECG pattern (negative T wave and prolonged QT interval regardless of the causative mechanism) in patients with stunned myocardium [55] and T wave inversion and QT interval prolongation and life-threatening arrhythmias in patients with Takotsubo cardiomyopathy [56].

T wave inversion on the ECG (in leads V1–V3) may be also due to the persistence of the juvenile pattern of repolarization [57]. In athletes, T wave inversion in, at least, 2 adjacent leads is a common ECG abnormality of hypertrophic and arrhythmogenic right ventricular cardiomyopathy, the leading cause of sudden cardiac death in athletes [9, 57]. T wave inversion in anterior leads is more prevalent in athletes of Afro-Caribbean origin compared to white competitors and represents an ethnic variant of athlete's heart, especially when associated with convex ST segment elevation [44]. T wave inversion in the lateral leads may represent a sign of hypertrophic cardiomyopathy, requiring further cardiovascular evaluation and follow-up [44]. T wave inversion and ST segment depression, pathological Q waves, and ventricular preexcitation are considered major, training-unrelated ECG abnormalities in athletes [9].

Not only do negative T waves have prognostic value, but also* positive T waves* in lead aVR in 169 consecutive patients, with acute ST segment elevation myocardial infarction, who underwent primary percutaneous coronary intervention, were associated with an increased in-hospital cardiovascular mortality [58].

*The T wave alternans*, a repeating ABABAB variability pattern in the morphology and amplitude of the T wave, reflects heterogeneity of repolarization and arises from beat-to-beat alteration of action potential duration at the level of cardiac myocytes, serves as a mechanism of arrhythmogenesis, and expresses vulnerability to lethal ventricular arrhythmias [59]. Despite the name “T wave alternans” (TWA), it may also involve the ST segment and U wave. Visual inspection may rarely reveal TWA due to the low amplitude, but computerized filtering and advanced analysis of the ECG enable its assessment. Ambulatory ECG recording-based TWA can be analyzed in 24-hour Holter recordings with daily activity, assessing the peak TWA value, and permits visual inspection in order to verify the presence and amplitude of TWA [60].

T wave axes were also associated with increased mortality [61, 62].

Inverted T waves, prolonged TpTe intervals, positive T waves in aVR, and T wave axes are predictors of cardiovascular mortality.

## 8. Spatial Angles

The ECG* spatial T axis* and the* spatial and frontal plane angles between the QRS and T vectors* are strong independent predictors of coronary heart disease and total mortality [14]. Several publications revealed an increased mortality risk related to wide QRS/T angles [63, 64]. The spatial angle between Tpeak and normal repolarization reference vector was the strongest predictor of coronary heart disease mortality and sudden cardiac death in men, and T amplitude in aVR and T onset and Tpeak vector magnitude ratio in women in a population with cardiovascular disease (CVD) in the Atherosclerosis Risk in Communities (ARIC) study [65]. T aVR amplitude negativity reduced to less than 150 *μ*V was the strongest mortality predictor in all subgroups [65]. Other ventricular repolarization variables, such as the spatial angles between mean QRS and T vectors, and between Tpeak and normal R reference vectors, respectively, and T in V1 were independent predictors of coronary heart disease and sudden cardiac death [66].

The spatial angle between mean QRS and T vectors is a measure of the overall deviation angle between depolarization and repolarization sequences, and the spatial angle between Tpeak and normal R reference vectors is a measure of deviation of the repolarization direction from the reference direction during regional repolarization of the left ventricular lateral wall [66]. Altered direction of the repolarization sequence may reflect anterior subepicardial myocardial ischemia, and the increased spatial angle between Tpeak and normal R reference vector may reflect a subclinical coronary heart disease and increased dyssynchrony of repolarization [66]. The spatial angle between Tpeak and normal R reference vectors and T aVR amplitude were also associated with coronary heart disease mortality in 52,994 postmenopausal women from the Women's Health Initiative study [67].

The most important limitation of the mentioned indices is the need of programmed analysis of electronic ECG signals and reconstruction of *X*, *Y*, and *Z* orthogonal leads [14].

## 9. Premature Ventricular Contractions (PVCs) and Nonsustained Ventricular Tachycardia

Ventricular arrhythmias are life-threatening arrhythmias, commonly associated with coronary heart disease. Morphology of the premature ventricular beats can have prognostic value.* Notching* of the premature ventricular complexes represent myocardial scar [32], and myocardial infarction was diagnosed if a QR or QRS pattern was present with a Q wave exceeding 40 ms [68].

Episodes of* nonsustained ventricular tachycardia* may be detected in Holter electrocardiographic monitoring of patients with hypertrophic cardiomyopathy, in patients with a high risk of subsequent sudden cardiac death [69].

Premature ventricular contractions are not infrequent in athletes without any heart disease [70]. Sudden deaths in athletes are frequently triggered by intense physical activity during competitions, due to underlying cardiac ion channelopathies, hypertrophic cardiomyopathy, arrhythmogenic right ventricular cardiomyopathy, or cardiac arrhythmogenic remodeling due to long training, which justifies clinical concern [71–73]. Two or more PVCs per 10 seconds and atrial or ventricular arrhythmias are considered training-unrelated pathological ECG findings in athletes according to the latest guidelines [13]. Exercise induced tachyarrhythmia in highly trained athletes without a structural heart disease, including premature ventricular beats, was benign and not associated with fatal events or the development of heart disease in a study enrolling 5,011 athletes, with a follow-up of 7 years [73].

## 10. ECG Ventricular Hypertrophy

Several ECG criteria were described for* left ventricular hypertrophy*, such as the classical voltage criteria, including the Sokolow-Lyon criteria: sum of the S wave in V1 and of the R wave in V5 or V6 ≥ 35 mm [74], and combined criteria, including the Cornell product [75], the Romhilt-Estes scoring system [76], and the Mazzaro score [77].

ECG left ventricular hypertrophy criteria, despite low sensitivity, are valuable risk markers in the epidemiology and prevention of cardiovascular disease [78], especially sudden cardiac death. In hypertensive patients, left ventricular hypertrophy is a sign of target organ damage and strongly predicts sudden cardiac death, myocardial infarction, congestive heart failure, and stroke [79, 80]. Both Cornell product and Sokolow-Lyon LVH were risk factors for stroke in a Japanese general population, even in normotensive subjects [81]. Heart rate, Q waves, and Cornell voltage-duration product were independently associated with cardiovascular death in 1,473 patients with asymptomatic aortic stenosis, followed up for 4.3 years [82].

The LIFE study (Losartan Intervention for Endpoint Reduction in Hypertension) showed that ECG LVH defined by Cornell product criteria, Sokolow-Lyon voltage criteria, or ECG strain improved prediction of cardiovascular events and that regression of ECG LVH during antihypertensive treatment was associated with better outcome, independent of blood pressure reduction [83].

Discrepancies between ECG and cardiac magnetic resonance imaging LVH results are related to several electrocardiographic variables, such as prolongation of QRS duration (false-negative LVH-ECG status), minor STT abnormalities, or major electrocardiographic abnormalities (false-positive LVH-ECG status) [84].

*Physiologic left ventricular hypertrophy* due to sustained physical exertion increases QRS voltages according to the classical hypothesis on the ECG left ventricular hypertrophy. The type of exercise, isometric or isotonic, is known to influence wall thickness [85]. A decrease in QRS magnitude after 21 months of competitive aerobic gymnastics training was found in 12 female athletes, despite increase in height and body mass index at the end of the training period, in agreement with the voltage deficit hypothesis during the early stages of left ventricular hypertrophy [86]. Current ECG guidelines for athletes consider isolated R and S wave amplitudes exceeding traditional criteria for LVH, as a physiological response to exercise training (physiologic LVH), and they do not predict cardiovascular mortality [9, 13]. The main problem in elite athletes is the differentiation between physiologic left ventricular hypertrophy, due to sustained physical training, and pathologic LVH due to hypertrophic cardiomyopathy, associated with sudden cardiac death [87]. It has been demonstrated that the new Refined Criteria for ECG Interpretation in athletes improved specificity in diagnosis of hypertrophic cardiomyopathy for black and Arabic athletes [13]. It has been recently shown that intense endurance exercise causes* right* and not left* ventricular dysfunction*, with apparent complete short-term recovery, yet structural and functional changes of the right ventricle were evident in some athletes [72]. A study including 40 athletes, studied at baseline, immediately after an endurance race and one week after the race, showed that the acute reduction in right ventricular function increased with race duration and correlated with biomarkers of myocardial injury (B-type natriuretic peptide and troponin I) and there were no changes of the left ventricular function [72]. Considering that greater reductions in right ventricular function occurred in athletes competing for a longer duration, it was suggested that the heart has a finite capacity to maintain an increased cardiac output [72]. Training level influences also the reduction in right ventricular function and myocardial injury [72]. In endurance athletes, stroke volume increases to more than 75% of maximal oxygen consumption, secondary to increased diastolic filling and ventricular emptying, increasing cardiac output for several hours [88]. The right ventricle may be overwhelmed due to prolonged, excessive volume overload, resulting in right atrial and ventricular dilation and right ventricular dysfunction; repetitive transitory chamber dilatation due to extreme exhaustion may lead to appearance of patchy cardiac fibrosis, as a substrate for ventricular tachyarrhythmia and nonischemic sudden arrhythmic death [6, 88]. Sustained increase of cardiac output, leading to transitory chamber dilation and patchy cardiac fibrosis in predisposed persons, is termed Phidippides cardiomyopathy, in other words, cardiac arrhythmogenic remodeling due to prolonged strenuous exercise [6]. Voltage criteria for right ventricular hypertrophy were identified exclusively in male athletes, due to more profound cardiac adaptations than in females, and were not associated with cardiac pathology in asymptomatic athletes [9]. Patients with normal ECGs or with isolated QRS voltage criteria for left ventricular hypertrophy may have hypertrophic cardiomyopathy, but a less severe phenotype, associated with a lower arrhythmic risk [89].

ECG LVH is a predictor of sudden cardiac death, myocardial infarction, congestive heart failure and stroke, and regression of LVH due to therapy improves outcomes. Sustained clinical exertion causes physiologic left ventricular hypertrophy, which needs to be differentiated from hypertrophic cardiomyopathy, and impairs right ventricular function. Isolated QRS voltage criteria for left or right ventricular hypertrophy are common, training-related ECG abnormalities in athletes [9].

## 11. Conclusions

Simple ECG markers are valuable noninvasive methods in identifying patients at increased risk of poor outcome, suggesting an important role in risk stratification in the general population and routine ECG screening in asymptomatic individuals, identifying patients who could benefit of more intensive management of cardiovascular risk factors and preventive interventions. The main ECG parameters associated with cardiovascular mortality were the P wave (duration, interatrial block, and deep terminal negativity of the P wave in V1), prolonged QT and Tpeak-Tend intervals, QRS duration and fragmentation, bundle branch block, ST segment depression and elevation, inverted T waves, spatial angles between QRS and T vectors, premature ventricular contractions, and ECG hypertrophy criteria. Clear cut-off values are still needed for most of the mentioned ECG variables.

ECG is also a valuable tool in risk-benefit estimation of physical exercise and preparticipation screening of athletes, considering that sports activity may trigger fatal cardiovascular events, including sudden cardiac death in individuals with predisposing cardiovascular factors. ECG can contribute to screening and early diagnosis of silent cardiovascular diseases, risk stratification and prevention of sudden cardiac death through lifestyle changes, and restriction of competitive sports activity, drugs, and implantable defibrillators. A single tracing is virtually diagnostic in a high proportion of cases, and serial tracings may confirm the diagnosis. Unfortunately, ECG may show no diagnostic feature in several cardiac conditions, especially when the patients do not have any symptoms and many abnormal patterns may be nonspecific, requiring additional testing. Besides false-positive and false-negative results, several pitfalls may result in ECG interpretation.

All repolarization related variables require also further attention in the evaluation of possible toxic drug effects. Standard 12-lead ECG merits further larger studies, in order to identify new ECG variables and to evaluate their importance in cardiovascular disease prognosis.
